# Phenolic Content of Brown Algae (Pheophyceae) Species: Extraction, Identification, and Quantification

**DOI:** 10.3390/biom9060244

**Published:** 2019-06-22

**Authors:** Ivana Generalić Mekinić, Danijela Skroza, Vida Šimat, Imen Hamed, Martina Čagalj, Zvjezdana Popović Perković

**Affiliations:** 1 Department of Food Technology and Biotechnology Faculty of Chemistry and Technology, University of Split, R. Boškovića 35, 21000 Split, Croatia; danci@ktf-split.hr; 2 University Department of Marine Studies, University of Split, R. Boškovića 37, 21000 Split, Croatia; vida@unist.hr (V.Š.); imen_hh@yahoo.fr (I.H.); martina.cagalj@hotmail.com (M.Č.); zvjezdana.popovic@unist.hr (Z.P.P.)

**Keywords:** brown algae, biologically active compounds, phenolics, phlorotannins, extraction, quantification

## Abstract

Over the last few decades, isolations and chemical characterizations of secondary metabolites with proved biological activities have been of interest for numerous research groups across the world. Phenolics, as one of the largest and most widely distributed group of phytochemicals, have gained special attention due to their pharmacological activity and array of health-promoting benefits. Reports on phenolic potentials of marine algae, especially brown algae (Pheophyceae) that are characterized by the presence of phlorotannins, are still scarce. The aim of this review paper is to provide an overview of current knowledge about phenolic potential of different brown algae species (74 species from 7 different orders). Studies on brown algae phenolics usually involve few species, thus the focus of this review is to provide information about the phenolic potential of reported algae species and to get an insight into some issues related to the applied extraction procedures and determination/quantification methods to facilitate the comparison of results from different studies. The information provided through this review should be useful for the design and interpretation of studies investigating the brown algae as a source of valuable phytochemicals.

## 1. Introduction

In the last decade the marine ecosystem has attracted researchers’ attention as many organisms possess or produce compounds with high biological activity. Some excellent candidates for research are macroalgae (seaweeds), a heterogeneous group of photosynthetic organisms other than the land plants inhabiting marine waters [1,2,3,4,5,6,7]. The total world production of commercial aquatic plants, including macroalgae, reached 31.2 million tonnes in 2016, with aquaculture macroalgae accounting for 96.5% [8]. Macroalgae are frequently exposed to the adverse environmental conditons and the in vivo damaging effects on them are not visible, what implies to their ability to generate various metabolites (enzymes, pigments, polysaccharides, vitamins, phenolics, tocopherols, phospholipids, etc.) that protect them from external factors [9,10,11,12,13,14].

Macroalgae form the basic biomass in the intertidal zone and they lack many of the distinct organs found in terrestrial plants. There are more than 19,000 different species of macroalgae [15,16] frequently classified on the basis of their photosynthetic pigments but also by differences in many ultra-structural and biochemical features including type of storage material, cell wall composition, presence/absence of flagella, ultrastructure of mitosis, connections between adjacent cells, and the fine structure of the chloroplasts [17,18,19].

They are usually divided into three divisions: green (phylum Charophyta and phylum Chlorophyta), red (phylum Rhodophyta), and brown (phylum Ochrophyta, class Phaeophyceae) [16]. The class of brown algae contains about 265 genera and 2040 species; about 95% of these species are marine organisms that are most prevalent in cold to temperate waters [16,20]. However, only three orders, namely Laminariales, Fucales and Dictyotales, and among them species of *Laminaria*, *Ecklonia*, *Undaria*, *Himanthalia,* and *Dictyota,* have been extensively investigated for their phytochemical composition. Numerous factors such as species, season, age, geographical location, and environmental conditions influence the differences in the algae phytochemical profile and content. All studies on brown algae report important levels of phenolics, characterized by extremely high biological activity, and comparatively, a higher content and more active antioxidants than green and red algae [2,19,21].

This manuscript reviews and compares published literature from 2000 dealing with the extraction of phenolic compounds from brown macroalgae and for that purpose electronic databases (Scopus and ScienceDirect) were searched (by the keywords). The focus of the paper was to get an insight into phenolic content of various brown algae (Pheophyceae) species from different orders and geographical origins as well as examine the impact of experimental conditions, such as type of solvent, solid-to-solvent ratio, temperature and time of extraction, and application of some novel extraction techniques on their phenolic profile.

## 2. Brown Algae Phenolics

The phenolics are secondary metabolites defined as aromatic benzene ring compounds possessing one or more hydroxyl groups bonded directly to an aromatic ring, including their functional derivatives. These phytochemicals display a wide variety of structures, from simple moieties to polymers with high molecular weight and biogenetically they arise from two main primary synthetic pathways; the shikimate pathway and the acetate pathway [22,23,24,25].

While polyphenolic compounds from terrestrial plants are usually derived from gallic and ellagic acid, the phenolics from marine macroalgae vary from simple molecules such as phenolic acids to highly complex compounds called phlorotannins (PHT). This subgroup of tannins is formed by polymerization of phloroglucinol (1,3,5-trihydroxybenzene, PG) units. Although the exact biosynthetic pathways for PHT are unknown, it has been proposed that PG is formed via the acetate-malonate (polyketide) pathway [2,3,26,27,28]. PHT are secondary metabolites restricted to brown seaweeds which are known to exist in soluble (occurring in cytoplasm or within cell organelles) or in cell wall-bound forms like other tannins. The existing data on phlorotannins are based solely on its soluble forms stored in physodes, which are highly mobile organelles observed in the cytoplasm. Also, it has been suggested that these components become primarily constituents of the cell wall and adhesives when physodes fuse with the membrane and the phlorotannins are secreted into the cell wall where they create complexes with alginic acid [29,30]. Therefore, it is obvious that phlorotannins play multiple roles in brown algae, both at the cellular and at the organismal level [26,30,31,32,33]. Phlorotannins are important in all stages of the algae, from its early developmental stages to adult plants. As all other phenolics, PHT present a heterogeneous and high molecular weight group of compounds, with content up to 20% in dry algae [19,34]. Based on the nature of the structural linkages between PG units and the number and distribution of hydroxyl groups, PHT can be divided into four major subclasses: phlorethols and fuhalols (ether linkages, aryl-ether bonds and para- and ortho-arranged ether bridges containing one additional –OH group in every third ring), fucols (phenyl linkages, aryl–aryl bonds), fucophlorethols (ether and phenyl linkages), and eckols (dibenzo [1,4] dioxin linkage, at least one three-ring moiety with a dibenzodioxin element substituted by a phenoxyl group at C-4) [3,26,34].

Being the dominant algae phenolics, PHT have chemical properties and putative physiological roles similar to those of tannins in vascular plants. Besides being primary components of algae cell walls, PHT play a prominent role as chemical defense against herbivores, bacteria, and fouling organisms. They may serve to deter grazers, absorb harmful UV radiation, and be involved in the protection against oxidative damage, in a heavy metal resistance mechanism, etc. [27,35]. Although these organelles may occur in most tissues of brown algae, a number of authors have noted a particular abundance of physodes in outer tissues (epidermal, outer cortical, apical, and meristematic cells) which points out that phenolic compounds have a prominent role in the thalus protection from excess irradiation and damage by UV radiation [30,31]. Algae are exposed to extreme environmental conditions (UV radiation, nutrient availability, salinity, temperature, high oxygen concentrations, etc.) that induce formation of oxidizing agents, such as free radicals and other reactive species, however they do not suffer any serious structural and photodynamic damage during metabolism [28]. The reason could be the production of various metabolites and among them phenolics are known as extremely good reducing agents and free radical scavengers that can potentially interact with biological systems. Different studies evidenced the significant positive pharmacological and nutraceutical properties of PHT as well as their potential application in different industries (food, pharmaceutical, cosmetic, etc.) [2,27,30,33,36,37,38].

### 2.1. Extraction

An insight into the phenolic potential of brown algae includes the review of phenolics from 74 algae species from 7 different orders (Desmarestiales, Dictyotales, Ectocarpales, Fucales, Laminariales, Sphacelariales, and Tilopteridales). Although PHT are distributed in different brown algae species, their concentration is highly variable among different taxa, being reported the highest in Fucoid species [26]. Furthermore, the content of PHT in algae is influenced by different abiotic and biotic factors such as species, plant stage, size, age and reproductive status, location, depth, nutrient enrichment, salinity, light intensity exposure, ultraviolet radiation, intensity of herbivory, and time of collection. Therefore, the full exploitation of algal diversity and complexity requires knowledge of environmental impacts and an understanding of biochemical and biological variability [26,27].

Table 1, Table 2, Table 3 and Table 4 summarize the results of studies on phenolic potential of different brown algae species of different geographical origin: Algeria [39], Australia [28,40,41,42], Brazil [43], China [44,45], Denmark [46], France [47,48,49,50], Iceland [51], India [12,37], Iran [52], Ireland [53,54,55,56,57,58], Japan [59,60,61,62], Korea [63], Lebanon [64], Malaysia [65,66,67], New Zealand [42], Portugal [50], South Korea [68], Spain [69,70,71,72,73], Thailand [74,75], and Tunisia [4,76,77]. The investigated species belong to different orders: Desmarestiales (1 species), Dictyotales (12 species), Ectocarpales (1 species), Fucales (42 species), Laminariales (14 species), Sphacelariales (3 species), and Tilopteridales (1 species). Besides the influence of the harvest location, the content of phenolics also showed diurnal and seasonal variations in kelps of the same populations that is extremely important for determination of their optimal collecting periods [26,39,40].

Generally, the analysis of phenolics is influenced by their nature, the extraction procedure employed, sample particle size, storage conditions and time, as well as the used assay for their determination and presence of interfering substances in extracts such as waxes, fats, pigments, etc. [24]. These compounds are very difficult to isolate quantitatively due to their large size and molecular weight, structural similarity, and tendency to react with other compounds. The results among studies are hard to properly compare due to different extraction conditions and result expression [30]. There is no single extraction protocol for preparation of phenolic extracts from algal material, since studies are dealing with various extraction parameters such as type of solvent [57,58,61,62,65,71,73], solid-to-solvent ratio [56,57], temperature [56,57,62], time of extraction [62], and application of some novel extraction techniques [40,41,42,45,48,50,56,73,75]. The solvent extraction is the most commonly utilized. It is time-consuming and requires large amounts of solvents. Furthermore, the applied procedure depends on the solubility of the desired compounds; while polar compounds, like PHT, solubilize very easily in highly polar solvents such as water, alcohols and acetone; lipophilic compounds (like vitamin E and carotenoids) can only be extracted using non-polar (or low-polar) solvents like hexane (Hex) and chloroform (Chl). The chemical nature of the compounds is a restricting factor in finding a solvent extraction system that is suitable for the extraction of all classes of phenolics or a specific class of them. Their solubility is governed by the type of solvent used, degree of polymerization and their interactions with other food constituents what leads to formation of insoluble complexes [24]. PHT usually form complexes with different components of algae cell walls; therefore, the protocols to obtain extracts enriched in PHT should be optimized to improve their extractability [49]. Also, as PHT are prone to oxidation, usually potassium disulfite or similar agents are added to the extraction solvent in order to decrease the rate of oxidation [26].

In most extraction procedures, highly hydrophilic phlorotannins, ethanol (EtOH) and methanol (MeOH) aqueous mixtures are used [3,12,37,41,44,45,47,48,49,54,61,64,65,69,71,72,73], with the most expressed tendency for EtOH in cases when extracts are prepared for food, pharmaceutical or cosmetic use.

Chew et al. [65] reported the content of phenolic compounds in *Padina antillarum* methanolic extract and in aqueous mixtures (20% and 50%). The highest content was obtained in 50% MeOH while 2-fold lower content was obtained in pure (100%) methanolic extract. López et al. [71] reported totally contrary results for *Halopteris scoparia* extracts where the highest yield of phenolics was detected in MeOH extracts, and the lowest in its 50% aqueous mixture. Otero et al. [73] also reported higher content of phenolics (more than 2-fold) in EtOH aqueous mixture (50%) than in EtOH only, same as Machu et al. [62] in 80% MeOH extracts of *Undaria pinnatifida* where the content was even 4.5-fold higher in comparion to the pure MeOH extract. On the other hand, Del Pilar Sanchez-Camargo et al. [49] investigated water, ethanolic and 50% EtOH extracts of *Sargassum muticum* prepared by the same protocols at three different temperatures and in all cases the highest content of phenolics was detected in EtOH extracts, while the lowest was found in water extracts.

The reported results regarding the use of water as an extraction solvent for algae phenolics are different among studies. Machu et al. [62] reported water to be the best solvent for the extraction of phenolics from *Eisenia bicyclis*, *Sargassum fusiforme*, *Saccharina japonica,* and *Undaria pinnatifida* in comparison to the aqueous MeOH and ACE extracts. The lowest phenolic content in algal water extracts, among other studied solvents, were also reported for extracts of *Dictyopteris polypodioides* [76], *Ecklonia cava* [63], *Fucus vesiculosus* [50,51], and *Sargassum muticum* [49]. Tierney et al. [56] reported the lowest yield of extracted phenolic compounds in water extracts of *Fucus spiralis* obtained by the conventional extraction and pressurized liquid extraction (PLE), that was not confirmed in the case of *Pelvetia canaliculata* as well as in *Ascophyllum nodosum* extracts prepared using PLE. The use of acidified solvents in the study of Kadam et al. [58] resulted in lower content of phenolics in comparison to the pure water that can be attributed to the fact that acidic solvents at high temperatures (70 °C) may be detrimental to phenolic compounds althought it could be expected that low pH medium in combination with high temperature could result in hydrolysis of the complex phenolic structures into more simple ones. Júnior et al. [43], Wang et al. [51] and Tierney et al. [56] reported that the use of ACE as extraction solvent gave the highest total yield of extracted phenolics probably due to inhibiting interactions between tannins and proteins during extraction or even by breaking hydrogen bonds between tannin-protein complexes [27]. In the study conducted by Airanthi et al. [61], the phenolic contents of *Eisenia bicyclis*, *Kjellmaniella crassifolia,* and *Alaria crassifolia* extracts obtained using different solvents have been reported and the obtained result on the basis of dry seaweed matter as well as per gram of extract. According to the results expressed per 100 g of dry seaweed it can be seen that the highest content of phenolics was detected in methanol extracts (from 72 to 87 mg PCE/100 g) and those results show correlation with tested antioxidant properties. In the case when the results are expressed per g of methanolic extracts the highest concentrations were detected in *E. bicyclis* and *A. crassifolia* hexane extracts and *K. crassifolia* chloroform extract, and the mentioned correlation was not confirmed [61]. Chakraborty et al. [78] reported the lowest yield of phenolics in hexane extracts of *Turbinaria conoides* and *Turbinaria ornata* same as Otero et al. [73] in *Laminaria ochroleuca* extracts. Relatively high content of total phenols was also detected in algal ethyl acetate (EtOAc) extracts [58,76,77,78]. As PHT are polar compounds, the exteremly high results for phenolic content obtained for extracts prepared using non-polar or low-polar solvents could be questionable especially because the widelspread Folin–Ciocalteu method is non-specific so the reagent could be affected and/or reduced by many interfering substances (e.g., sugars, proteins, aromatic amines, or organic acids). In case of alcoholic solvents, methanolic extracts gave higher yields of phenolics in comparison to ethanolic extracts. This was by Airanthi et al. [61] on *Alaria crassifolia* and *Himanthalia elongata*, Heffernan et al. [57] on *Fucus serratus* and *Laminaria digitata*, López et al. [71] on *Halopteris scoparia* and Rattaya et al. [76] on *Sargassum polycystum* and *Turbinaria ornata*.

The conventional solvent extraction is considered time-consuming and expensive, causing degradations of the products. Due to health concerns and environmental issues, “green” approaches and some novel techniques of extraction have been developed and used for algal extraction. Among them, solid-phase extraction (SPE), supercritical fluid extraction (SFE), ultrasound-assisted extraction (UAE), microwave-assisted extraction (MAE), and pressurized liquid extraction (PLE) were tested and described [26,28,40,41,42,45,48,49,56,58].

PLE, also called pressurized solvent extraction (PSE) or accelerated solvent extraction, (ASE) utilizes elevated pressures (10 to 15 MPa) and temperatures (50 to 200 °C) in combination with low solvent volumes to extract compounds in a short time (minutes as opposed to hours). In MAE technique, the extraction occurs because of changes in the cell structure caused by electromagnetic waves. However, the main disadvantage of PLE, as well as MAE, is the possible degradation of thermolabile compounds at higher temperatures. These problems could be avoided by the use of UAE that utilizes sonic energy to disintegrate the cell structure and release bioactive compounds in a short time [79,80]. SFE technique is an extraction method that uses fluids in their supercritical conditions, with temperature and pressure above their critical point what cause their liquid-like characteristics (increased mass transfer due to low viscosity and higher diffusion coefficient). The most used solvent is CO_2_ and this technique is efficient in the extraction of nonpolar compounds but the extraction of polar substances can be enhanced by adding small amounts of polar co-solvents such as ethanol or methanol. The usefulness of SFE strongly depends on the type of compounds to be extracted as there is an exteremly tendency to nonpolar compounds so this method is rarely used for the extraction of polar phenolics [81,82].

Studies of Zubia et al. [48], Plaza et al. [70], Kadam et al. [58], Tierney et al. [56], Del Pilar Sánchez-Camargo et al. [49], Dang et al. [29,40,41], Magnusson et al. [42], Yuan et al. [45], and Otero et al. [73] reported on the use of these techniques for the extraction of biologically active phenolics from algal materials whatresulted in extracts containing higher amount of phenolics. Kadam et al. [58] reported higher yield of phenolics in extracts of *Ascophyllum nodosium* and *Laminaria hyperborea* obtained by UAE in comparison to the conventional extraction. Similarly, the higher content of phenolics in UAE extracts was reported by Dang et al. [29,41] on *Hormosira banksii* and *Sargassum vestitum*. The use of MAE in preparation of MeOH extracts of *Ascophyllum nodosium*, *Saccharina japonica*, *Lessonia nigrecens,* and *Lessonia trabeculate* resulted in higher content of phenolics than in extracts obtained by shaking at RT for 4 h [83]. Dang et al. [40] also reported higher yield of phenolics in MAE extracts of *S. vestitum* than in those obtained by UAE or conventional procedure, same as Magnusson et al. [42] on MAE extracts of *Carpophyllum flexuosum*. Otero et al. [73], Del Pilar Sanchez-Camargo et al. [49], Tierney et al. [56], Plaza et al. [70], and Zubia et al. [48] investigated applications of PLE in extraction of phenolics from algal biomass. All studies reported higher content in extracts obtained by application of this novel technique. Plaza et al. [70] investigated the influence of extraction temperature on phenolic content and concluded that higher temperature (200 °C vs. 100 °C) resulted with higher content of phenolics in all investigated extracts; 2.5-fold higher concentration in *Saragassum vulgare*, 5.5-fold higher concentration in *Saragassum muticum*, 7-fold higher concentration in *Cystoseira abies-marina*, and more than 17.7-fold higher concentration in *Undaria pinnatifida*. Del Pilar Sanchez-Camargo et al. [49] also investigated the impact of temperature (50, 125, and 200 °C) influenced by the solvent (W, EtOH and 50% EtOH) on the content of the extracted phenolic compounds. A higher temperature resulted with higher yield of phenolics in water and 50% EtOH extracts, while a slight decrease of phenolics was detected in EtOH extracts which contained the highest concentration of phenolics among all studied extracts. Machu et al. [62] investigated the phenolic content of nine algal food products after treatment with different extraction methods and according to their report the extraction using hot water (80 °C, for 10 min) was the best solvent for all analyzed brown seaweed samples (*Hizikia fusiformis*, *Eisenia bicyclis*, *Laminaria japonica*, and *Undaria pinnatifida*). Although the extraction time for these extracts was very short, they were significantly richer in phenolics in comparison to 80% methanolic extracts prepared at 70 °C during 1 h (from 4.5 to 21-fold higher phenolic content) or in pure methanol by maceration during 24 h at room temperature (23 °C) (from 5.7 to 19-fold higher phenolic content). Otero et al. [73] also studied efficiency of the extraction from *Laminaria ochroleuca* at different temperatures (80 and 160 °C) using different solvents (Hex, EtOH and 50% EtOH). Ethanolic extracts contained higher amounts of phenolic compounds probably due to use of higher temperatures but also the polarity of the solvent. Also, use of 50% EtOH in comparison to the pure EtOH resulted in 2-fold higher concentration of phenolics. Furthermore, solid-to-solvent ratio in the extraction procedure also varies and it ranges from 1:5 to 1:100 in different studies (Table 1). As this parameter has a great influence on the final concentration of phenolics, prepared liquid extracts are usually evaporated or lyophilized and the concentration of phenolics is expressed on the basis of dry algal mass (per g of dw) or dry extract (per g) enabling the comparison of the results among different studies.

The extraction temperature also varies; from room temperature (RT) usually applied in conventional extractions [39,42,43,61] to significantly high temperatures applied in novel extraction techniques [47,53,62]. The application of high temperatures (above 60 °C) in the extraction procedure mays be questionable for two reasons: i) the susceptibility of phenolic compounds to thermal degradation [24,45], ii) causing hydrolysis of complex phlorotannin compounds into simplex compounds that can generally increase the total phenolic content. Generally, the application of higher temperatures in experiments using PLE resulted in extracts with higher yield of phenolics [49,70,73]. However, Heffernan et al. [57] reported lower content of phenolics in extracts of *Fucus serratus* prepared at RT by shaking for 24 h in comparison to those obtained at 60 °C. Also, the extraction time ranged from few minutes up to few days depending on the chemical composition of the prepared extracts. As expected, prolonged contact (extraction) time usually results with higher content of phenolics as reported by Zaragoza et al. [47] on *Fucus vesiculosus*.

### 2.2. Quantification and Identification

Different techniques are also used for detection, identification, and quantification of PHT in brown algae, such as colorimetric methods, high-performance liquid chromatography, microscopy, capillary electrophoresis, and quantitative proton nuclear magnetic resonance. Most commonly, total PHT content is quantified by colorimetric methods, generally used for detection of phenolics, like Folin–Ciocalteu, Folin–Denis, and Prussian blue assays. The use of this assay conceals the chemical diversity of individual compounds present but because these compounds are reactive, polar, and structurally related to each other, there is a lack of more sophisticated analysis methods [24,27,30,84].

Among all mentioned assays for quantification of phenolics, the Folin–Ciocalteu method is the most widely used. This assay indirectly measures the content of compounds that can react in a redox type reaction with a Folin–Ciocalteu reagent. The disadvantage of this method is the interference of other non-phenolic reducing substances with the determinations. Due to the polarity of phenolics, their high concentrations that were reported to be obtained by non-polar solvents resulted probably from the non-specificity of the reagents and the effect of interfering (reducing) substances (like pigments, sugars, proteins, aromatic amines, organic acids, inorganic substances, and different metal chelators) that are also present in the extracts. As the prepared extracts are complex mixtures of compounds from different classes that were also soluble in the used solvent, additional steps are required to remove unwanted non-phenolic compounds or to purify the isolates [24]. These observations could be applied to the results of studies reporting high content of phenolics in non-polar organic solvent extracts.

Another problem regarding the analysis using the Folin–Ciocalteu assay is that scientists use different standard compounds for the expression of the results, additionally complicating their comparison among different studies. Due to the complexity of phenolics as well as the differences in the reactivity toward reagents used for their detection, it is very hard to find a specific and suitable standard for quantification [24]. Gallic acid equivalents (GAE) are widely used in the studies on terrestrial plants, but some studies are reporting the phenolic content in phloroglucinol equivalents (PGE) [4,12,42,53,58], pyrocatechol equivalents (PCE) [61], phlorotannin content (PTC) [44], catechin equivalents (CE) [71,75], etc. Aside of the result expressions in mg of (some) standard compounds, authors are reporting the content of phenolics (phlorotannins) on the basis of dry algae extracts or dry algae weight (plant material).

To summarize, if all the results for total phenolics are expressed in mg of PGE/g, the highest concentrations are reported in species from the same order (genus Fucus): *F. vesiculosus* (277 mg PGE/g) [47], *F. serratus* (240 mg PGE/g) [56] and *F. spiralis* (204 mg PGE/g) [56]. The results in PGE expressed on the dry algal basis (per g dw) also vary, and they ranged from 0.34 in *A. nodosum* [58] up to 114 mg PGE/g dw in *Carpophyllum flexuosum* [42]. The results for phenolics in brown alge that are expressed in mg of gallic acid equivalents (mg GAE) are also reported in different studies. These data per g of dry extract ranged from 0.063 mg in *Sargassum binderi* [74] to 303.0 mg in *Ecklonia stolonifera* [60], or from 1.23 mg in *H. scoparia* [71] to 406 mg in *Cystoseira crinita* [77] when expressed per g of dry algae mass. Airanthi et al. [61] reported from 1.87 mg PCE/g in *E. bicyclis* ethanolic extracts to 31.33 mg/g in methanolic extract of *Staphanocystis hakodatensis*. Chkhikvishvili and Ramazanov [69] reported the content of phenolic substances in 14 algal species on the dry algae weight basis (in %), and the richest in phenolics were brown algae *Cystoseira foeniculacea*, *Labophora variegata*, and *Stypopodium zonale*. Among algae from the order Dictyotales (Table 1) the highest content of phenolics has been found in *Dictyota dichotoma* (35.23 mg PGE/g) [66], *Stypopodium zonale* (1.22% dw) [69] and *Padina* sp. (124.65 mg GAE/g) [28]. In the order Fucales (Table 2) the most investigated species were *Ascophyllum nodosum* [51,56,58,83], *Fucus serratus* [48,51,55,69], *Fucus vesiculosus* [46,47,51,55,84], and *Himanthalia elongata* [53,54,61,72]. The reported species were also the richest in phenolics: 159 mg PGE/g (51) and 1.4 mg GAE/g dw [83] in *Ascophyllum nodosum*, 240 mg PGE/g [51] and 81.93 mg GAE/g [57] in *Fucus serratus* and 277 mg PGE/g [47] and 165 mg GAE/g dw [48]. Also, high content of phenolics was detected in *Fucus spiralis* (from 90.79 to 204.40 mg PGE/g) [59] and *Cystoseira crinite* (from 261.53 to 406.22 mg GAE/g dw) [77]. Among Laminariales the most potent species are *Ecklonia kurome* (97 mg PGE/g) [59], *Ecklonia bicyclis* (192.8 mg GAE/g) [62] and *Laminaria ochroleuca* (173.65 mg GAE/g) (Table 3) [73].

**Figure 1:**
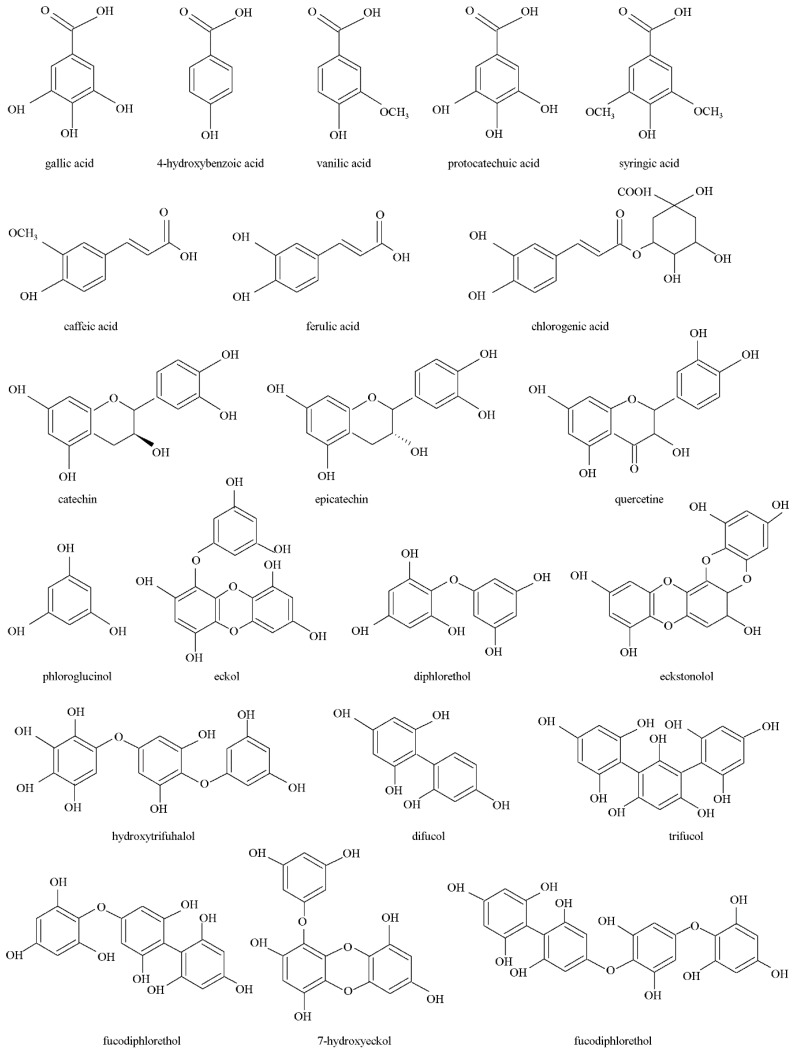
Chemical structures of phenolic compounds from marine brown algae.

Various chromatographic techniques have been employed for separation, preparative isolation, purification, identification, and quantification of individual phenolic compounds from various plant materials, but still a small number of studies deals with the individual phenolic compounds from brown algae. The high solubility of PHT facilites their qualitative and quantitative analysis using high-performance liquid chromatography (HPLC) in combination with mass spectrometry (MS) or nuclear magnetic resonance (NMR) [32]. Still, the identification and quantification of PHT is usually performed by reversed phase high performance liquid chromatography (RP-HPLC) with MeOH/acetonitrile and water (buffer) solvent combinations and the detection in the UV range of the spectrum [26]. Despite extensive research on the plant phenolics using chromatography techniques, analogous studies for brown algal PHT are still rare. Among all studies reported in this review paper, only few of them analyzed the presence of individual phenolic compounds. Figure 1 presents the chemical structures of major phenolic compounds detected in brown algae species.

Polyphenolic compounds such as flavonols/glycosides of flavonol, chroman ring containing phenolics gallocatechin, gallate of catechin, and epicatechin were abundant in seaweeds [11]. López et al. [71] identified 14 phenolics in extracts of *Halopteris scoparia* with variations among investigated extracts regarding the solvent used. In their study, gallic acid, catechin, epicatechin, and gentisic were found in the highest concentration. Kumar et al. [74] detected and quantified 17 different phenolic compounds in algal tissues with catechin and epicatechin being the most abundant, while among compounds from the sub-group of phenolic acids high concentrations of gallic, chlorogenic, syringic and gentisic acids were found. Yuan et al. [45] detected 17 peaks, and the major components of *A. nodosum*, *Saccharina japonica*, *Lessonia trabeculata* and *Lessonia nigrescens* extracts include phenolic acid, phlorotannin and catechin derivatives.

Wijesinghe et al. [85] confirmed the presence of PHT triphloroethol-A, eckol, dieckol, and eckstolonol in investigated *Ecklonia calva* samples, while Machu et al. [62] confirmed the presence of gallic acid (*E. bicyclis, H. fusiformis*), 4-hydroxybenzoic acid (*U. pinnatifida*), epicatechin (*E. bicyclis*, *Sargassum fusiforme, Saccharina japonica*), catechin gallate (*E. bicyclis, S. japonica*) and epigallocatechin (*S. japonica*).

Belda et al. [72] identified and quantified 11 phenolic compounds in *H. elongata* among which the PG was the most abundant as well as gallic acid from the subgroup of phenolic acids. As reported by Chakraborty et al. [38], phenolic acids were the predominant phenolics in EtOAc fraction of *Anthophycus longifolius* while epicatechin gallate and catechin were detected in MeOH extracts. Furthermore, the presence of phenolic acids and quercetine was confirmed in EtOAc extract of *Saragassum plagiophyllum*.

## 3. Conclusions

Considering all the reports given in this review paper it is clear that the studies conducted on different algal species vary in the extraction protocols and expression of the results, which makes the comparison of the results from different studies extremely hard. The establishment of the standardized extraction protocol for preparation of algal phenolic extracts is unlikely, thus the possible solution may be expressing the results using the same standard compound. Despite this, brown algae should be considered as new and valuable source of biologically active compounds with many possible applications in the food, pharmaceutical, and cosmetic industries. These species are still being investigated and screened for biomolecules, especially phenolic group of PHT that are not found in terrestrial sources. Therefore, their isolation, identification and pharmacological characterisation are still relatively new scientific areas where new and sustainable trends should be followed.

## Figures and Tables

**Table 1:** Overview of the phenolic content of brown algae from order Dictyotales.

Algae Species	Collecting Location	Collecting Period	Solvent	Plant: Solvent Ratio	Extraction Mode	Total Phenolic Content	Reference
*Dictyota ciliolata*	Spain	-	70% MeOH	-	-	0.08% dw	[69]
*Dictyota dichotoma*	France	May 2007	DCM, MeOH	-	PLE, 75 °C, 1500 psi	18.8 mg PGE/g	[48]
	India	December	MeOH	1:5	48 h, RT	0.02 mg PGE/g	[12]
	Malaysia	-	MeOH	1:10	72 h, RT	35.23 mg PGE/g	[66]
*Dictyota sp. 1*	Spain	-	70% MeOH	-	-	0.03% dw	[69]
*Dictyota sp. 2*	Spain	-	70% MeOH	-	-	0.001% dw	[69]
*Dictyopteris polypodioides*	Tunisia	July 2006	W	1:10	24 h, 4 °C	33.8 mg GAE/g dw	[77]
			Chl			84.96 mg GAE/g dw	
			EtOAc			60.96 mg GAE/g dw	
	Spain	-	70% MeOH	-	-	0.09% dw	[69]
*Lobophora variegata*	Spain	-	70% MeOH	-	-	1.20% dw	[69]
*Padina antillarum*	Malaysia	October–December 2005	20% MeOH	1: 50	Shaking	20.4 mg GAE/g dw	[65]
			50% MeOH			24.3 mg GAE/g dw	
			MeOH			12.4 mg GAE/g dw	
*Padina pavonica*	Tunisia	July 2015	50% EtOH	1:5	Shaking, 30 min, 50 °C	7.06 mg PGE/g dw	[4]
	Spain	-	70% MeOH	-	-	0.69% dw	[69]
	Lebanon	2010	MeOH	1:10	Shaking, 72 h, RT	10.55 mg GAE/g	[64]
*Padina sp.*	Australia	March 2016	70% EtOH	1:50	UAE, 1 h, 150 W, 30 °C	124.65 mg GAE/g	[28]
	Malaysia	-	MeOH	1:10	72 h, RT	33.11 mg PGE/g	[66]
*Spatoglossum schroederi*	Brasil	December 2011– January 2012	MeOH	1:20	Shaking, 24 h, RT	11.75 mg GAE/g	[43]
			ACE			14.10 mg GAE/g	
			Chl			6.84 mg GAE/g	
*Stypopodium zonale*	Spain	-	70% MeOH	-	-	1.22% dw	[69]
*Zonaria tournefortii*	Algeria	December 2013, June and September 2014	W	1:100	Shaking, 1 h, RT	0.78 mg GAE/g	[39]
	Spain	-	70% MeOH	-	-	1.06% dw	[69]

DCM—dichloromethane, MeOH—methanol, ACE—acetone, EtOH—ethanol, Chl—chloroforme, EtOAc—Ethyl acetate, RT—room temperature, GAE—gallic acid equivalents, PGE—phloroglucinol equivalents, UAE—ultrasound assisted extraction, PLE—pressurized liquid extraction, dw—dry weight.

**Table 2:** Overview of the phenolic content of brown algae from order Fucales.

Algae Species	Collecting Location	Collecting Period	Solvent	Plant: Solvent Ratio	Extraction Mode	Total Phenolic Content	Reference
*Anthophycus longifolius*	India	-	MeOH	1:6	3 h, 45 °C	41.11 mg GAE/g	[38]
*Ascophyllum nodosum*	Ireland	-	60% MeOH	1:15	3 h, 40 °C	4.5 mg GAE/g dw	[55]
	Iceland	May 2007	W	1:20	Shaking, 24 h, RT	138 mg PGE/g	[51]
			70% ACE			159 mg PGE/g	
	Ireland	May 2014	W	1:20	UAE, 35.61 Wcm^−2^, 15 min	0.16 mg PGE/g dw	[58]
			0.1 M HCl			0.13 mg PGE/g dw	
			W		Shaking, 150 min, 70 °C	0.17 mg PGE/g dw	
			0.1 M HCl			0.11 mg PGE/g dw	
		March 2010	W	1:20	Shaking, RT	70.48 mg PGE/g	[56]
			80% EtOH	1:10		66.26 mg PGE/g	
			80% ACE	1:10		155.95 mg PGE/g	
			W	-	PLE, 120 °C, 1500 psi	93.44 mg PGE/g	
			80% EtOH	-	PLE, 100 °C, 1000 psi	101.30 mg PGE/g	
			80% ACE	-	PLE, 60 °C, 1000 psi	127.37 mg PGE/g	
	China	-	70% MeOH	1:10	MAE, 110 °C, 2.45 GHz, 15 min	1.40 mg GAE/g dw	[82]
			70% MeOH	1:10	Shaking, 4 h, RT	0.51 mg GAE/g dw	
*Bifurcaria bifurcata*	France	September 2007	MeOH	-	PLE, 75 °C, 1500 psi	9.6 mg PGE/g	[48]
*Carpophyllum flexuosum*	New Zealand	July 2014	MeOH, ACE	1:8	Shaking, RT	86 mg PGE/g dw	[42]
			W	1:30	MAE	114 mg PGE/g dw	
*Carpophyllum plumosum*	New Zealand	July 2014	MeOH, ACE	1:8	Shaking, RT	75 mg PGE/g dw	[42]
*Cystophora subfarcinata*	Australia	November 2014	MeOH, ACE	1:8	Shaking, RT	22 mg PGE/g dw	[42]
*Cystoseira abies-marina*	Spain	-	W	1:10	PLE, 20 min, 100 °C, 1500 psi	6.81 mg GAE/g	[70]
					PLE, 20 min, 200 °C, 1500 psi	48.09 mg GAE/g	
*Cystoseira compressa*	Spain	-	70% MeOH	-	-	4.83% dw	[69]
*Cystoseira crinita*	Tunisia	June 2007	Chl	-	24 h	402.44 mg GAE/g dw	[78]
			EtOAc			406.22 mg GAE/g dw	
			MeOH			261.53 mg GAE/g dw	
*Cystoseira foeniculacea*	Spain	-	70% MeOH	-	-	2.16% dw	[69]
*Cystoseira sedoides*	Tunisia	July 2015	50% EtOH	1:5	Shaking, 30 min, 50 °C	26.45 mg PGE/g dw	[4]
*Cystoseira tamariscifolia*	France	April–June 2007	DCM, MeOH	-	PLE, 75 °C, 1500 psi	10.91 mg PGE/g	[48]
*Fucus ceranoides*	France	March 2006	DCM, MeOH	-	PLE, 75 °C, 1500 psi	54.7 mg PGE/g	[48]
*Fucus serratus*	France	March 2007	DCM, MeOH	-	PLE, 75 °C, 1500 psi	28.2 mg PGE/g	[48]
	Iceland	March 2007	W	1:20	Shaking, 24 h, RT	169 mg PGE/g	[51]
			70% ACE			240 mg PGE/g	
	Ireland	-	60% MeOH	1:15	3 h, 40 °C	4.0 mg GAE/g dw	[55]
		2011	W (cold)	1:10	Shaking, 24 h, RT	81.93 mg GAE/g	[57]
			80% EtOH	1:20		75.96 mg GAE/g	
			80% MeOH	1:20		80.70 mg GAE/g	
			W (hot)	1:10	Shaking, 24 h, 60 °C	79.49 mg GAE/g	
*Fucus spiralis*	Spain	-	70% MeOH	-	-	2.17% dw	[69]
	Ireland	May 2010	W	1:20	Shaking, RT	90.79 mg PGE/g	[56]
			80% EtOH	1:10	Shaking, RT	124.30 mg PGE/g	
			80% ACE	1:10	Shaking, RT	204.40 mg PGE/g	
			W	-	PLE, 120 °C, 1500 psi	130.58 mg PGE/g	
			80% EtOH	-	PLE, 100 °C, 1000 psi	142.81 mg PGE/g	
			80% ACE	1:20	PLE, 60 °C, 1000 psi	187.55 mg PGE/g	
*Fucus vesiculosus*	France	-	30–35% EtOH	1:10	Shaking, 4 h, RT	277 mg PGE/g	[47]
			50–75% EtOH		Shaking, 2 h, RT	163 mg PGE/g	
	Ireland	-	60% MeOH	1:15	3 h, 40 °C	2.5 mg GAE/g dw	[55]
	Iceland	March 2007	W	1:20	Shaking, 24 h, RT	17.6 mg PGE/g	[51]
			70% ACE			24.2 mg PGE/g	
	Denmark	September	W	1:20	Shaking, 24 h, 125 rpm, 20 °C	134 mg GAE/g dw	[46]
			80% EtOH			165 mg GAE/g dw	
	Portugal	January 2016	W	1:20	RT, 24 h	14.8 mg GAE/g	[50]
			W	1:20	90 °C, 30 min	17.4 mg GAE/g	
			80% EtOH	1:20	RT, 24 h	56.6 mg GAE/g	
			70% ACE	1:20	RT, 24 h	39.1 mg GAE/g	
*Halidrys siliquosa*	France	May 2007	DCM, MeOH	-	PLE, 75 °C, 1500 psi	16.0 mg PGE/g	[48]
*Hormosira banksii*	Australia	March 2016	70% EtOH	1:50	Shaking, 12 h, 30 °C	16.21 mg GAE/g	[41]
					UAE, 60 min, 150 W, 30 °C	23.12 mg GAE/g	[41]
						158.82 mg GAE/g	[28]
*Himanthalia elongata*	Spain	-	60% MeOH	1:5	Shaking, 2 h, 60 °C	5.48 mg GAE/g	[72]
	Ireland	June, September 2008	60% MeOH	1:10	Shaking, 2 h, 40 °C	151.33 mg GAE/g	[53]
		-	60% MeOH	1:5	Shaking, 2 h, 40 °C	52.50 mg GAE/g	[54]
*Kjellmaniella crassifolia*	Japan	-	MeOH	1:10	24 h, dark, RT	9.90 mg PCE/g	[61]
			EtOH			8.01 mg PCE/g	
			ACE			11.75 mg PCE/g	
			Chl			17.82 mg PCE/g	
			EtOAc			14.12 mg PCE/g	
			Hex			16.67 mg PCE/g	
*Pelvetia canaliculata*	Ireland	-	60% MeOH	1:15	3 h, 40 °C	4.0 mg GAE/g dw	[55]
		May 2010	W	1:20	Shaking, RT	41.13 mg PGE/g	[56]
			80% EtOH	1:10	Shaking, RT	40.07 mg PGE/g	
			80% ACE	1:10	Shaking, RT	168.82 mg PGE/g	
			W	-	PLE, 120 °C, 1500 psi	73.65 mg PGE/g	
			80% EtOH	-	PLE, 100 °C, 1000 psi	61.89 mg PGE/g	
			80% ACE	-	PLE, 60 °C, 1000 psi	68.24 mg PGE/g	
*Saragassum aquifolium*	Australia	March 2016	70% EtOH	1:50	UAE, 1 h, 150 W, 30 °C	67.78 mg GAE/g	[28]
		November 2014	MeOH, ACE	1:8	Shaking, RT	2 mg PGE/g dw	[42]
*Sargassum binderi*	Thailand	May 2007	W	1:10	Shaking, 72 h, 25 °C	0.267 mg GAE/g	[75]
			EtOH			0.063 mg GAE/g	
*Sargassum boveanum*	Iran	May 2006	W	1:100	15 min, 121 °C	17.0 mg CE/g dw	[52]
			EtOH	1:12.5	Shaken, 4.5 h, 37 °C	0.9 mg CE/g dw	
*Sargassum desfontainesii*	Spain	-	70% MeOH	-	-	1.68% dw	[69]
*Sargassum flavicans*	Australia	August 2014	MeOH, ACE	1:8	Shaking, RT	15 mg PGE/g dw	[42]
*Sargassum fusiforme*	China Japan	April-June 2014	30% EtOH	1:50	Shaking, 30 min, 25 °C	880 mg PTC/g	[44]
		-	100% MeOH	1:100	Shaking, 24 h, 23 °C	6.0 mg GAE/g	[62]
			70% ACE	1:100	Shaking, 30 min, 30 °C	13.1 mg GAE/g	
			80% MeOH	1:100	Shaking, 1 h, 70 °C	9.5 mg GAE/g	
			MeOH:W:HAc (30:69:1)	1:100	Shaking, 50 min, 70 °C	26.9 mg GAE/g	
			W	1:100	Shaking, 10 min, 80°C	34.5 mg GAE/g	
*Sargassum furcatum*	Spain	-	70% MeOH	-	-	2.97% dw	[69]
*Sargassum horneri*	Japan	2007	MeOH	1:10	24 h, dark, RT	9.90 mg PCE/g	[61]
*Sargassum linearifolium*	Australia	March 2016	70% EtOH	1: 50	UAE, 1 h, 150 W, 30 °C	47.06 mg GAE/g	[28]
*Sargassum muticum*	Spain	-	W	1:10	PLE, 20 min, 100 °C, 1500 psi	10.73 mg GAE/g	[70]
					PLE, 20 min, 200 °C, 1500 psi	58.67 mg GAE/g	
	France	July 2011	W	-	PLE, 20 min, 1500 psi, 50 °C	29.61 mg GAE/g	[49]
			EtOH			94.20 mg GAE/g	
			50% EtOH			58.10 mg GAE/g	
			W		PLE, 20 min, 1500 psi, 125 °C	52.26 mg GAE/g	
			EtOH			93.84 mg GAE/g	
			50% EtOH			76.62 mg GAE/g	
			W		PLE, 20 min, 1500 psi, 200 °C	69.31 mg GAE/g	
			EtOH			93.16 mg GAE/g	
			50% EtOH			82.22 mg GAE/g	
*Saragassum polycystum*							
*Sargassum plagiophyllum*	India	-	MeOH	1:6	3 h, 45 °C	7.48 mg GAE/g	
*Sargassum podacanthum*	Australia	March 2016	70% EtOH	1: 50	UAE, 1 h, 150 W, 30 °C	48.13 mg GAE/g	[28]
*Sargassum polycystum*	Thailand	-	MeOH	1:30	Shaking, 30 min, RT	0.59 mg CE/g dw	[76]
			EtOH			0.32 mg CE/g dw	
	Malaysia	-	50% EtOH	1:10	Shaking, 2 h, 65 °C	0.37 mg GAE/g dw	[67]
	Malaysia	-	MeOH	1:10	72 h, RT	45.16 mg PGE/g	[66]
	India	-	MeOH	1:6	3 h, 45 °C	8.71 mg GAE/g	[38]
*Sargassum vestitum*	Australia	March 2016	70% EtOH	1: 50	MAE	58.20 mg GAE/g	[40]
					Shaking, 12 h, 30 °C	40.31 mg GAE/g	
					UAE, 1 h, 30 °C	48.45 mg GAE/g	
					UAE, 1 h, 150 W, 30 °C	141.91 mg GAE/g	[28]
*Sargassum vulgare*	Spain	-	W	1:10	PLE, 20 min, 100 °C, 1500 psi	26.43 mg GAE/g	[70]
					PLE, 20 min, 200 °C, 1500 psi	70.86 mg GAE/g	
	Lebanon	2010	MeOH	1:10	Shaking, 72 h, RT	12.71 mg GAE/g	[64]
*Sirophysalis trinodis*	Australia	August 2014	MeOH, ACE	1:8	Shaking, RT	25 mg PGE/g dw	[42]
	India	December	MeOH	1:5	48 h, RT	0.15 mg PGE/g	[12]
			80% EtOH			62 mg PGE/g	
*Stephanocystis hakodatensis*	Japan	2007	MeOH	1:10	24 h, dark, RT	31.33 mg PCE/g	[61]
*Turbinaria conoides*	Thailand	May 2007	W	1:10	Shaking, 72 h, 25 °C	1.12 mg GAE/g	[75]
			EtOH			0.09 mg GAE/g	
	India	-	MeOH	1:10	3 h, 40–45 °C	16.64 mg GAE/g	[79]
			Hex			19.26 mg GAE/g	
			DCM			51.47 mg GAE/g	
			EtOAc			105.97 mg GAE/g	
*Turbinaria ornata*	India	-	MeOH	1:10	3 h, 40–45 °C	3.42 mg GAE/g	[79]
			Hex			1.07 mg GAE/g	
			DCM			12.72 mg GAE/g	
			EtOAc			69.63 mg GAE/g	
	Thailand	-	MeOH	1:30	Shaking, 30 min, RT	2.18 mg CE/g dw	[76]
			EtOH			1.25 mg CE/g dw	
*Zonaria tournefortii*	Algeria	December 2013, June and September 2014	W	1:100	Shaking, 1 h, RT	0.78 mg GAE/g	[39]
	Spain	-	70% MeOH	-	-	1.06% dw	[69]

DCM—dichloromethane, MeOH—methanol, ACE—acetone, EtOH—ethanol, Hex—hexane, Chl—chloroforme, EtOAc—Ethyl acetate; HAc—Acetic acid; RT—room temperature, PCE—pyrocatechol equivalents; GAE—gallic acid equivalents, PGE—phloroglucinol equivalents, PTC—phlorotannin content, CE—catechin equivalents, MAE—microwave assisted extraction, UAE—ultrasound assisted extraction, PLE—pressurized liquid extraction, dw—dry weight.

**Table 3:** Overview of the phenolic content of brown algae from order Laminariales.

Algae Species	Collecting Location	Collecting Period	Solvent	Plant: Solvent Ratio	Extraction Mode	Total Phenolic Content	Reference
*Laria crassifolia*	Japan	2007	MeOH	1:10	24 h, dark, RT	5.94 mg PCE/g	[61]
			EtOH			7.21 mg PCE/g	
			ACE			12.44 mg PCE/g	
			Chl			12.93 mg PCE/g	
			EtOAc			16.11 mg PCE/g	
			Hex			21.13 mg PCE/g	
*Alaria esculenta*	France	May 2007	DCM, MeOH	-	PLE, 75 °C, 1500 psi	20.3 mg PGE/g	[48]
*Ecklonia cava*	South Korea	May 2004	MeOH	1:50	72 h, 25 °C	82.99 mg GAE/g dw	[68]
	Korea	-	W	-	Boiling	20.7 mg PGE/g	[63]
			30% EtOH	-	2 h, 50 °C	45.3 mg PGE/g	
			80% EtOH	1:50	Shaking, 2 h, RT	28.96 mg GAE/g	[84]
*Ecklonia kurome*	Japan	March 2006	W	1:20	Shaking, 20 min, 75 °C	97 mg PGE/g	[59]
			80% EtOH			62 mg PGE/g	
*Ecklonia stolonifera*	Japan	March 2006	W	1:20	Shaking, 20 min, 75 °C	74 mg PGE/g	[59]
			80% EtOH		Shaking, 20 min, 75 °C	73 mg PGE/g	
		-	W	1:4	-	82.13 mg GAE/g	[60]
			EtOH		-	303.0 mg GAE/g	
*Ecklonia bicyclis*	Japan	-	MeOH	1:100	Shaking, 24 h, 23 °C	9.5 mg GAE/g	[62]
			70% ACE		Shaking, 30 min, 30 °C	84.1 mg GAE/g	
			80% MeOH		Shaking, 1 h, 70 °C	143.2 mg GAE/g	
			MeOH:W:HAc (30:69:1)		Shaking, 50 min, 70 °C	192.8 mg GAE/g	
			W		Shaking, 10 min, 80 °C	192.6 mg GAE/g	
			MeOH	1:10	24 h, dark, RT	7.77 mg PCE/g	[61]
			EtOH			1.87 mg PCE/g	
			ACE			4.11 mg PCE/g	
			Chl			14.41 mg PCE/g	
			EtOAc			9.13 mg PCE/g	
			Hex			16.75 mg PCE/g	
*Laminaria digitata*	Ireland	June, September 2008	60% MeOH	1:10	Shaking, 2 h, 40 °C	37.66 mg GAE/g	[53]
		2011	W (cold)	1:10	Shaking, 24 h, RT	2.24 mg GAE/g	[57]
			80% EtOH	1:20		1.39 mg GAE/g	
			80% MeOH	1:20		2.93 mg GAE/g	
			W (hot)	1:10	Shaking, 24 h, 60 °C	5.06 mg GAE/g	
		-	60% MeOH	1:5	Shaking, 2 h, 40 °C	35.80 mg GAE/g	[54]
*Laminaria hyperborea*	Ireland	-	60% MeOH	1:15	3 h, 40 °C	1.5 mg GAE/g dw	[55]
		May 2014	W	1:20	US, 35.61 Wcm^−2^, 15 min	0.37 mg PGE/g dw	[58]
			0.1 M HCl			0.34 mg PGE/g dw	
			W		Shaking, 2.5 h, 70 °C	0.36 mg PGE/g dw	
			0.1 M HCl			0.35 mg PGE/g dw	
*Laminaria ochroleuca*	Spain	August 2017	Hex	1:20	PLE, 10 min, 1450 bar, 80 °C	6 mg GAE/g	[73]
			EtOH		PLE, 10 min, 1450 bar, 160 °C	83 mg GAE/g	
			50% EtOH		PLE, 10 min, 1450 bar, 160 °C	173.65 mg GAE/g	
*Lessonia nigrecens*	China	-	70% MeOH	1:10	MAE, 110°C, 2.45 GHz, 15 min	1.07 mg GAE/g dw	[45]
			70% MeOH	1:10	Shaking, 4 h, RT	0.78 mg GAE/g dw	
*Lessonia trabeculata*	China	-	70% MeOH	1:10	MAE, 110 °C, 2.45 GHz, 15 min	0.74 mg GAE/g dw	[45]
			70% MeOH	1:10	Shaking, 4 h, RT	0.50 mg GAE/g dw	
*Saccharina latissima*	France	April 2016	W	1:20	RT, 24 h	4.7 mg GAE/g	[50]
			W	1:20	90 °C, 30 min	7.8 mg GAE/g	
			80% EtOH	1:20	RT, 24 h	1.9 mg GAE/g	
			70% ACE	1:20	RT, 24 h	5.2 mg GAE/g	
	Ireland	June, September 2008	60% MeOH	1:10	Shaking, 2 h, 40 °C	66.75 mg GAE/g	[53]
		-		1:5	Shaking, 2 h, 40 °C	43.50 mg GAE/g	[54]
*Saccharina japonica*	Japan	-	100% MeOH	1:100	Shaking, 24 h, 23 °C	0.7 mg GAE/g	[62]
			70% ACE		Shaking, 30 min, 30 °C	8.8 mg GAE/g	
			80% MeOH		Shaking, 1 h, 70 °C	14.9 mg GAE/g	
			MeOH:W:HAc (30:69:1)		Shaking, 50 min, 70 °C	8.5 mg GAE/g	
			W		Shaking, 10 min, 80 °C	8.7 mg GAE/g	
	China	-	70% MeOH	1:10	MAE, 110 °C, 2.45 GHz, 15 min	0.73 mg GAE/g dw	[45]
			70% MeOH	1:10	Shaking, 4 h, RT	0.38 mg GAE/g dw	
*Undaria pinnatifida*	Spain	-	W	1:10	PLE, 20 min, 100 °C, 1500 psi	3.79 mg GAE/g	[70]
					PLE, 20 min, 200 °C, 1500 psi	67.11 mg GAE/g	
	Japan	-	MeOH	1:100	Shaking, 24 h, 23 °C,	1.3 mg GAE/g	[62]
			70% ACE		Shaking, 30 min, 30 °C	5.7 mg GAE/g	
			80% MeOH		Shaking, 1 h, 70 °C	5.9 mg GAE/g	
			MeOH:W:HAc (30:69:1)		Shaking, 50 min, 70 °C	3.7 mg GAE/g	
			W		Shaking, 10 min, 80 °C	8.6 mg GAE/g	

DCM—dichloromethane, MeOH—methanol, ACE—acetone, EtOH—ethanol, Hex—hexane, Chl—chloroforme, EtOAc—Ethyl acetate; HAc—Acetic acid; RT—room temperature, PCE—pyrocatechol equivalents; GAE—gallic acid equivalents, PGE—phloroglucinol equivalents, MAE—microwave assisted extraction, PLE—pressurized liquid extraction, dw—dry weight.

**Table 4:** Overview of the phenolic content of brown algae from other orders.

Algae Species	Order	Collecting Location	Collecting Period	Solvent	Plant: Solvent Ratio	Extraction Mode	Total Phenolic Content	Reference
*Asperococcus bullosus*	Ectocarpales	France	June 2007	DCM, MeOH	-	PLE, 75 °C, 1500 psi	11.1 mg PGE/g	[48]
*Cladostephus spongiosum*	Sphacelariales	Tunisia	July 2015	50% EtOH	1:5	Shaking, 30 min, 50 °C	10.91 mg PGE/g dw	[4]
*Desmarestia ligulata*	Desmarestiales	France	May 2007	DCM, MeOH	-	PLE, 75 °C, 1500 psi	12.2 mg PGE/g	[48]
*Halopteris scoparia*	Sphacelariales	Algeria	December 2012, June and September 2014	W	1:100	Shaking, 1 h, RT	1.05 mg GAE/g	[39]
		Spain	-	70% MeOH	-	-	0.16% dw	[69]
		Spain	March-April 2008	MeOH	1:15	Shaking, 2 h, RT	3.29 mg GAE/g dw	[71]
				EtOH			2.92 mg GAE/g dw	
				W			2.5 mg GAE/g dw	
				50% MeOH			1.23 mg GAE/g dw	
		Spain	March-April 2008	MeOH	1:15	Shaking, 2 h, RT	255.2 mg GAE/g dw	[71]
*Saccorhiza polyschides*	Tilopteridales	France	June 2007	DCM, MeOH	-	PLE, 75 °C, 1500 psi	16.6 mg PGE/g	[48]

DCM—dichloromethane, MeOH—methanol, EtOH—ethanol, RT—room temperature, GAE—gallic acid equivalents, PGE—phloroglucinol equivalents, PLE—pressurized liquid extraction, dw—dry weight.
